# Empirical distributions of traffic loads from one year of weigh-in-motion data

**DOI:** 10.1038/s41597-023-02212-0

**Published:** 2023-05-18

**Authors:** Iunio Iervolino, Georgios Baltzopoulos, Antonio Vitale, Antonio Grella, Giovanni Bonini, Antonio Iannaccone

**Affiliations:** 1 Dipartimento di Strutture per l’Ingegneria e l’Architettura, Università degli Studi di Napoli Federico II, Via Claudio 21, 80125 Naples, Italy; 2 MOVYON S.p.A., gruppo Autostrade per l’Italia, Via A. Bergamini 50, 00159 Rome, Italy; 3 Tangenziale di Napoli S.p.A., gruppo Autostrade per l’Italia, via G. Porzio 4, 80143 Naples, Italy

**Keywords:** Civil engineering, Statistics

## Abstract

In the state-of-the-art of structural engineering the actions for design or assessment of bridges should derive from a probabilistic (i.e., frequentist) characterization of the loads. Data from weigh-in-motion (WIM) systems can inform stochastic models for traffic loads. However, WIM is not widespread, and data of this kind are scarce in the literature and often not recent. Due to structural safety reasons, the 52 km long A3 highway in Italy, connecting the cities of Naples and Salerno, has been equipped with a WIM system which has been operational since the beginning of 2021. The system’s measurements of each vehicle transiting over the WIM devices, impede overloads on the many bridges featured in the transportation infrastructure. By the time of this writing the WIM system has seen one year of uninterrupted operation, collecting more than thirty-six million datapoints in the meantime. This short paper presents and discusses these WIM measurements, deriving the empirical distributions of traffic loads and making the original data available for further research and applications.

## Background & Summary

Verification of structural safety for bridges, under vehicle traffic, typically requires engineering calculations based on code-mandated conventional traffic loads. For existing bridges, such calculations are necessary to determine their operating safety margin, and to decide if traffic control or retrofitting measures are in order. State-of-the-art building codes^1,2^ adopt a semi-probabilistic approach to structural reliability^3,4^, which requires that the internal forces, induced on structural elements by these loads, reflect a given nominal exceedance probability in a time interval of interest. This requirement presupposes that conventional loads are calibrated against measurements of actual traffic. At the time of writing, this has emerged as an especially topical issue for Italy, where new rules and guidelines for structural safety checks to verify the operability of road bridges have been recently introduced^5^.

Direct traffic observations represent the straightforward approach to empirically analyze traffic loads with the purpose to derive load models, something that can be achieved via traffic micro-simulation^6^. Empirical measurement of traffic structural actions requires weigh-in-motion (WIM) systems to be continuously operational for long periods. Recognition of this fact, together with other considerations such as the need to protect infrastructure against overloading^7^ or to monitor pavement wear, have led to various initiatives in Europe and the U.S. to develop and install WIM stations along highway networks^8,9^. In the case of using WIM data to model traffic loads for safety verifications of specific existing bridges, availability of a structure-^10^ or network-specific WIM dataset, representative of local traffic composition, can be desirable^11^. This because local traffic may exhibit a systematic trend that needs to be captured, to develop models that are adequately representative of the circulating vehicle population.

In this context, as a case-study, one year’s worth of data from the WIM system installed on the A3 – *Napoli-Pompei-Salerno* highway in southern Italy are presented, analyzed, and made available for further research and applications^12^. The A3 is a busy transportation infrastructure connecting the two major cities of the Campania region. It directly links the ports of Naples and Salerno, which are among the most important in the Mediterranean Sea and provides access to the Sorrento and Amalfi coasts. Between 2021 and 2022 the WIM system recorded millions of vehicle passages (36,359,127), in the framework of a comprehensive traffic control campaign, meant to avoid overloads on the bridges. These data, whose amount has little precedent for the Italian highway network, have been made available by the highway operator to be analyzed in the study presented herein, where the empirical distributions of vehicle weight, axle number, geometry, and other features have been derived, and to be openly shared.

**Fig. 1:**
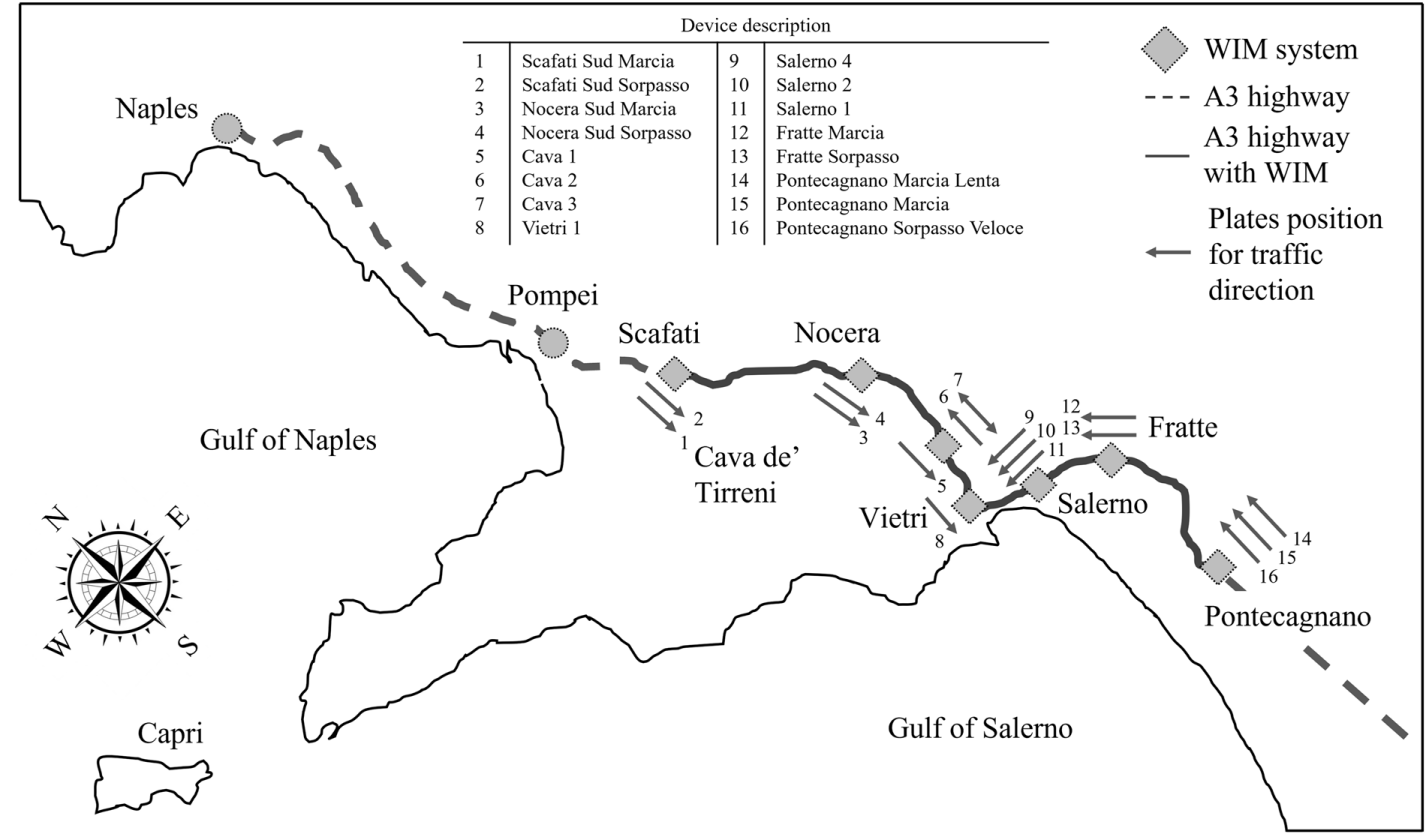
A3 highway trace (also designated E45 as part of the European transportation system) and location of the WIM devices, along with the associated “DataDescriptor” field codes.

The A3 Napoli-Pompei-Salerno highway is only 52 km long, schematically shown in Fig. 1, yet has played a decisive role in the development and integration of the thirty-odd municipalities situated in the areas around Mt. Vesuvius and the Sorrento peninsula since 1928, when the first tract, from Naples to Pompeii, was opened to traffic. With its second section, from Pompei to Salerno, completed in the early sixties, it provides services to about eight-hundred-thousand inhabitants and alleviates traffic in the eastern area of Naples’ hinterland. The highway connects an area which is densely populated and provides supply for the touristic mobility demand generated by archaeological, natural, and religious assets (Pompeii, Herculaneum, Sorrento, Positano, Vietri and Amalfi Coast, Sanctuary of Pompeii, etc.). It connects the ports of Naples and Salerno, which are among the most important for commodity shipping in Europe. It is part of the E45 European route and links to other major Italian highways A1 (*Autostrada del Sole*) to the north and A2 (*Autostrada del Mediterraneo*) to the south. In 2017 the average daily transits on the highway were about a hundred and seventy thousand, for a total of more than sixty million in one year. The 2022 data (to follow) show about thirty-six million transits in one year, that is a daily average of about one-hundred thousand.

The A3 has three lanes per traffic direction for most of its length, and part of the segment between Pompei and Salerno crosses several valleys, overlooking a majestic seacoast. Thus, the tract between the town of Cava de Tirreni and Salerno, features several viaducts of various structural typologies. Most noteworthy among those, from a structural engineering point of view, are reinforced concrete stiff deck and slender arch bridges, that is, bridges of the so-called *Maillart* type^13^.

Many of these bridges were built based on Swiss design in the fifties, and were chosen, among other reasons, for their elegant appearance, which fits the outstanding beauty of the Amalfi coast. However, they were designed for very different loads than currently prescribed and therefore may show some inadequacy with respect to the enforced standards^14^, which motivated the installation of a traffic control system, including WIM, discussed in the next section and shown in Fig. 1 for the first time (where arrows indicate the traffic direction at the installation), and which provided the data for this study^12^.

## Methods

The WIM system of the A3 highway is part of a traffic control system aimed at real-time monitoring of the traffic loads along its bridge infrastructure. The system enables to associate the weight of each vehicle transiting, or approaching, the A3 to its license plate, through a series of automatic license plate recognition (ALPR) cameras^15^. This allows to identify and monitor each vehicle’s path and offers the possibility of requesting, or enforcing, the rerouting of vehicles that do not comply with certain load-related criteria.

In fact, the system was put in place to monitor traffic in a segment of the A3, from Cava de Tirreni to Salerno. This was because several of the bridges on that segment had been found not fulfilling the safety verifications criteria according to the current Italian building code. This is not surprising, as most of these bridges were designed several decades ago, with standards, simplified design models, and loads, which can be now considered obsolete. This situation is not uncommon in Italy, in fact, it also represents the condition of most of the building stock^16^. Because of this, a set of guidelines was released in 2020, in the aftermath of the collapse of the Polcevera viaduct in Genova^17^, for the classification and management of risk, the assessment of safety and the monitoring of existing bridges, hereafter LL.GG.2020^5^. The intention behind the LL.GG.2020 guidelines was to enable temporary transit on structurally substandard bridges by limiting maximum allowable vehicle weight on the basis of structural monitoring and safety verifications. In this context, the level of traffic restriction depends not only on structural performance, but also on the level of traffic control imposed on the infrastructure of interest, that is, on the actual capacity of the network operator to prevent the transit of unauthorized vehicles. The largest admissible vehicle mass, according to the Italian transportation code, is 44 t (tons). In other words, only vehicles weighing 440 kN or less can circulate freely, without a special authorization.

To enable continued use of aging infrastructure, albeit with restrictions if the structural verifications demand it, the LL.GG.2020 guidelines prescribe traffic control that prohibits the transit of all overload vehicles, which are to be rerouted. Therefore, because, as mentioned, the A3 connects two busy seaports, it was in the interest of the former operator (*Autostrade Meriodionali* S.p.A., now superseded by SIS S.p.A.) to design and enforce a traffic control system to: (i) monitor all vehicles in transit on the route; (ii) identify vehicles with a mass exceeding 40 t; (iii) issue a timely warning to the drivers not to transit on the Cava de Tirreni-Salerno section; (iv) identify potential infractions of this traffic limitation and report the culprits to the police for immediate removal from the highway and administrative sanctions. This system integrates detection and alerting software algorithms with hardware elements such as cameras, active signalling systems and WIM technology. The system has been in continuous operation since its installation in early 2021, and the interested reader can find more details in material provided by the highway operator^18^ (https://www.stradeeautostrade.it/segnaletica-e-sicurezza/il-progetto-monitoraggio-overload-su-tratta-con-pese-dinamiche/). The raw data for this study was supplied by the former A3 highway operator, as part of a collaboration among the authors of this work. Others wishing to repeat the work or perform similar studies should approach the highway operator directly.

**Fig. 2:**
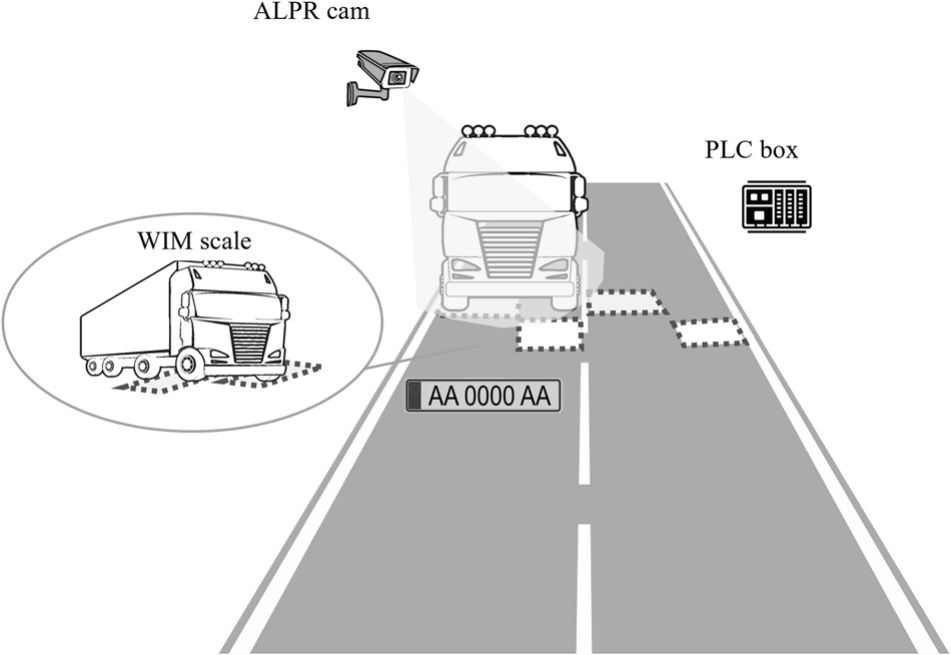
Illustrative description of the WIM system for measuring, among others, the weight of all vehicles passing from detection points.

The WIM devices (i.e., scales), that provide the data discussed in the following^12^, consist of austenitic stainless-steel plates equipped with optical fibre sensors^19^. As shown in the schematic representation of Fig. 2, these plates are embedded in the road pavement, so that a staggered pair of them are dedicated to cover an entire lane’s width, thus being able to intercept one semi-axle each, for any vehicle that stays exclusively within that lane while passing from the control point^20^. This enables not only the measurement of each axle’s load, but also inter-axle distance, as well as vehicle width. However, this also implies that measurements of vehicles that pass the control point astride two adjacent lanes must be flagged for possible inaccuracy of some of the aforementioned quantities (to follow). The weight measurements on the WIM scales have been certified for the dynamic load recorded to be within a ten percent tolerance of the corresponding static weight.

Seven of these WIM scales are located at the Cava de Tirreni, Vietri sul Mare, and Salerno interchanges, covering both traffic directions, as shown in Fig. 1. Four more scales are located between the interchanges at Scafati and Cava de Tirreni, monitoring southbound traffic, and another five where the A2 highway (between Salerno and Reggio Calabria) connects with A3. The latter are dedicated to monitoring northbound traffic, to prevent overloaded vehicles from entering the A3 from the A2 to the south. The main feature of the WIM system is real-time data updating and prompt communication with the highway’s central access system, via programmable logic controller (PLC) devices, whenever one of the devices detects a vehicle whose weight exceeds the permitted threshold, as defined above.

This short paper essentially provides empirical distributions of traffic loads and vehicle geometry characteristics based on one year of WIM data from a traffic control system for a 52 km long highway in Italy connecting, among other locations, two important ports in southern Italy. The provided data^12^ were continuously recorded from Feb. 1^st^ 2021 to Jan. 31^st^ 2022. Given that the focus of this dataset is on those parameters that are influential for the determination of structural actions on road bridges, such as inter-axle spacing and axle loads, especially for the heavier vehicles in transit, the data have to be cleared of possibly unreliable information and/or repeated observations of a single vehicle transit, to allow obtaining the empirical distributions of the vehicles’ main features.

Although there were some traffic limitations on the highway where the data have been collected, this quality control and filtering operations described below led to a population of about seventeen million transits. The resulting dataset can be used to derive sets of conditional empirical distributions useful for the calibration of bridge traffic loads, such as the distribution of axle loads and gross vehicle weight given number of axles, or vehicle weight category or type of axle grouping.

In this dataset^12^, three types of record quality are contemplated, tagged for brevity as OK, NL, and NR. NR stands for erroneous records, already flagged as such by the WIM data acquisition system, which are excluded from the dataset and further analyses. OK and NL can be considered usable, therefore only those are retained for subsequent elaboration and analysis. In fact, the acronym NL indicates a measurement that is not valid for legal purposes (i.e., imposing a fine for an infraction), even though it can be technically valid. An error code is associated to each NL-tagged data record, which is used to denote a traffic violation (e.g., speeding) or a warning of the WIM system for over-the-limit vehicle weight. NL-tagged records also include records based on partial information. This would be the case of vehicles that are not travelling exclusively within the confines of a single lane during the measurement, causing at least one axle to not pass over the WIM scale with both wheels. In this case, the axle’s load is estimated by duplicating the force measurement of the wheel that actually managed to activate the scale during transit.

In order to filter out spurious results, that have not been automatically flagged with an error code, some additional acceptance criteria were introduced, in accordance with international best practice^21,22^. These criteria consisted of parsing the data to detect null records or other logical inconsistencies in terms of vehicle dimensions, weight or velocity. More specifically, all records with total weight outside the interval [2 kN, 4000 kN], or with distances between two consecutive axles larger than 10 m or lower than 0.8 m were excluded from the dataset. Other grounds for exclusion from further analysis were records of vehicles with only a single axle, two-axle vehicles where one of the recorded axle loads was less than 20% of the vehicle’s total weight, an axle load less than 0.1 kN. Finally, vehicles with recorded lengths of less than 1.50 m and widths of less than 1 m were also excluded, most likely corresponding to motorcycles, for which reliable measurements are beyond the sensitivity of the WIM system.

Another type of result to be filtered out are multiple counts of the same vehicle on the same trip. To this end, all transits of the same vehicle detected within forty-six minutes of one another on more than one scale between the Pontecagnano and Scafati interchanges, in both directions, are only considered once (that being the time needed to make a round-trip between the two interchanges at an average speed of 60 km/h). Additionally, vehicles whose license plate was not recognized by the WIM system are also excluded. At the end of the data quality control filtering operations described above, the resulting number of records providing the basis for further elaboration, presented in the next section, is - as anticipated - about seventeen million.

## Data Records

The dataset are available at figshare^12^. Each of the sixteen devices comprising the WIM system, placed on various lanes of the A3 as described above, provides a data record for every vehicle transit detected. During the one-year operation, from Feb. 1^st^ 2021 to Jan 31^st^ 2022, about thirty-six million (36,359,127) records became available. The most relevant information provided in each record are: (1) date & time of measurement; (2) measurement quality and possible error code; (3) the vehicle’s total weight, length, and width; (4) the vehicle’s speed and acceleration; (5) the number of axles; (6) each axles’ load, width, and distance from other axles of the same vehicle; (7) left/right partitioning of each axle’s load. Axle indicates a metallic mechanical component connecting the wheels and for the purpose of these statistics all axles are initially considered individually, regardless of inter-axle distance; axle load refers to the sum of forces recorded by the WIM scales from the tires at both ends of an axle. In this context, vehicle length refers to the measured distance between the first and last axle. Another piece of information recorded by the WIM system, is each vehicle’s license plate number; although this does not constitute part of the data records provided, it is used for identifying each vehicle and filtering out multiple records of any vehicle on the same trip, as mentioned.

**Fig. 3:**
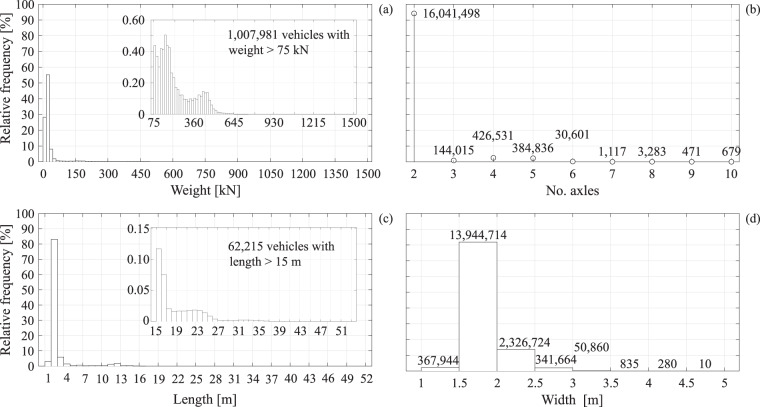
Marginal relative frequency distributions of specified vehicle’s features. (**a**) Marginal relative frequency distribution of the weight; (**b**) marginal relative frequency distribution of the number of axles; (**c**) marginal relative frequency distribution of the length; (**d**) marginal relative frequency distribution of the width.

For a graphical representation of the recorded data, Fig. 3 shows the marginal frequency distributions, in histogram format, of vehicle weight, number of axles, length and width. These histograms can be compiled after the raw recorded data have been appropriately filtered, as described in the Methods section. Therefore, the ordinates in the figure are the number of unique transits in each bin normalized by the total number of filtered observed data that amounts to just over seventeen million.

Recognizing that the modal value collects more than 80% of the data in three out of these four distributions, the readability of bins that count only a few thousand of data points is impaired in these graphs. For this reason, Fig. 3a is equipped with a magnified view of the data corresponding to vehicles with a weight greater than 75 kN, and similar for Fig. 3c, only this time for vehicle length exceeding 15 m. Panels (b) and (d) of the figure report the relative frequency of axle number per vehicle and for vehicle width, respectively. From these graphs, it can be seen that 94% of recorded vehicles travel on two axles and 82% of the same fall under the 1.5 m to 2.0 m width category. In terms of weight, the largest fraction is that of vehicles weighing between 10 kN and 40 kN, while in terms of length, most vehicles fall under the 2 m to 3 m bin. In other words, the majority of recorded traffic can be associated with passenger cars and light commercial vans, which is to be expected.

The dataset is provided under two alternative formats: MATHWORKS MATLAB® files (*.mat extension) and comma delimited text files (*.csv extension). In order to render file-size more manageable, the data have been divided over twelve files, each corresponding to one month’s worth of WIM records. Every file contains a table where each row corresponds to the record of a single vehicle transit. The column designations, indicating the type of information contained therein, are listed and explained here:

- *DeviceDescription*: designation identifying a specific WIM device, with a complete list of possible values provided in Fig. 1;
- *MeasureDate*: date (year/month/day format) and time of the vehicle record;
- *Outcomes*: type of record quality, with possible values for this field being OK or NL (see Methods section);
- *Weight*: gross vehicle weight in kilo-Newtons [kN] rounded to the nearest integer value;
- *Length*: length of the vehicle, measured as the distance between the first and last axle, in meters [m];
- *AxleNumber*: number of axles of the vehicle;
- *VehicleMeanWidth*: mean width of the vehicle in meters [m];
- *MinAxleDistance*: minimum inter-axle distance of the vehicle in meters [m];
- *MeanDistanc*e: mean distance between consecutive axles of the vehicle in meters [m];
- *AxleLoad*_*i*_: load transmitted by the i-th axle of the vehicle (i = 1,2,…,10) recorded by the WIM-system in kilo-Newton [kN]. Zero values indicate that the vehicle does not have as many axles as that;
- *MultipleAxleLoad*: vector of axle grouping type designations. Numerical symbol “1” indicates single axle, “2” indicates an axle that is part of a tandem axle system and “3” indicates an axle that is part of a tridem axle system, while numerical symbols ranging to “4” to “7” indicate an axle that is part of multi-axle system of four, five, six and seven axles. The i-th vector element corresponds to the AxleLoad_i_ column. For example, the vector [1 2 2 2 2 0 0 0 0 0] indicates a five-axle vehicle, with a single steering axle and two pairs of tandem axles, while the vector [1 3 3 3 1 2 2 0 0 0] would correspond to a seven-axle vehicle, with a single steering axle, three grouped into a tridem system followed by a single axle and then a tandem axle.

Note that in the above data fields, all length measurements have been rounded off to the nearest cm and that the gross vehicle weight has been rounded to the nearest integer value in kN. Although the axle load values are reported in kN units using two decimal digits, it is recalled that the WIM system in question is certified to provide axle loads within 10% of their static value.

Table 1 provides a synopsis of the data, divided per vehicle weight category according to the Italian transportation code. This categorization classifies vehicles with a total weight of less than 75 kN as *light*, between 75 kN and 260 kN as *medium*, between 260 kN and 440 kN as *heavy* and beyond 440 kN as *exceptional* vehicles. It should be highlighted that this last category actually comprises all vehicles who exceed the legal limit for free transit on the Italian highways, in terms of mass, thus requiring special authorization to do so.

**Table 1:** Number of vehicles in the sample classified per categories of Italian transportation code.

	Total	Weight ≤ 75 kN (Light)	75kN < Weight ≤ 260kN (Medium)	260kN < Weight ≤ 440kN (Heavy)	Weight > 440kN (Exceptional)
Transits	17,033,031	16,025,050	710,833	216,917	80,231
[%]	100	94	4.2	1.3	0.5

**Fig. 4:**
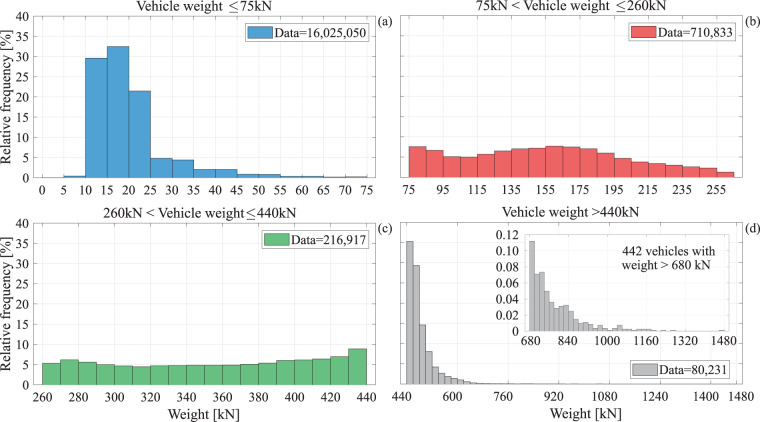
Gross vehicle weights. (**a**) Relative frequency distribution for light vehicles; (**b**) relative frequency distribution for medium vehicles; (**c**) relative frequency distribution for heavy vehicles; (**d**) relative frequency distribution for vehicles with mass greater than 44 t.

To enhance visualisation of the provided dataset, the following empirical frequency distributions were obtained for the vehicles: (1) gross vehicle weight, number of axles, vehicle length and width, given weight category, (2) gross vehicle weight given the number of axles, (3) maximum axle load given the number of axles, (4) axle-group load for single, tandem and tridem axles (5) mean inter-axle distance given the number of axles, (6) minimum axle distance given the number of axles. Thus, Fig. 4 shows the empirical distributions of vehicle weight within each of the four categories. Whilst light vehicles represent a 94% of the useable recorded data; with medium, heavy, and exceptional comprising the remaining 4.2%, 1.3% and 0.5%, respectively, statistics of the three heavier categories can, in fact, be more influential for traffic-load related engineering applications (to follow). This figure shows that vehicles within the 75 kN and 440 kN weight range follow a more uniform frequency distribution, unlike lighter vehicles where the distribution exhibits a distinct mode around 15 kN to 20 kN. On the other hand, the weight distribution of the exceptional vehicle category, starting at 440 kN, decreases sharply, reflecting the decreasing likelihood of increasingly massive objects being transported over the highway network, with the heaviest vehicle recorded tipping the scales at almost 1480 kN.

**Fig. 5:**
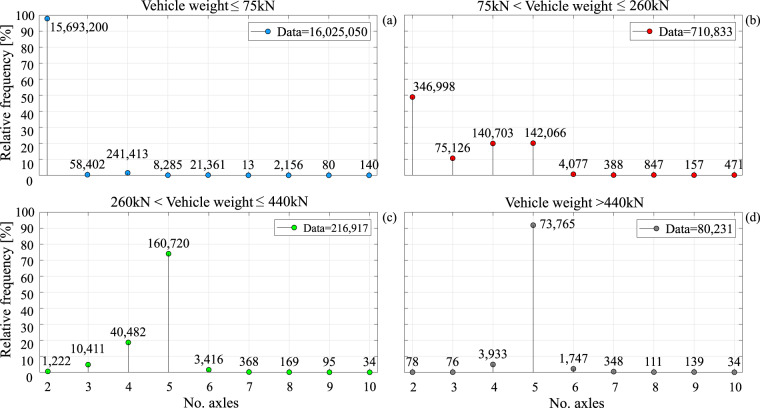
Number of axles per vehicle. (**a**) Relative frequency distribution of the number of axles conditioned to light vehicles; (**b**) relative frequency distribution of the number of axles conditioned to medium vehicles; (**c**) relative frequency distribution of the number of axles conditioned to heavy vehicles; (**d**) relative frequency distribution of the number of axles conditioned to vehicles with mass greater than 44 t.

Figure 5 shows the frequency distributions of axle number per vehicle, conditional to the vehicle’s weight range. All vehicles in the dataset have a minimum recorded number of axles equal to two, and a maximum of ten, as values outside this range have been considered potentially spurious and filtered out, as mentioned. As expected, the light-vehicle category is dominated by two-axle passenger and commercial vehicles, with 98% of the data points in panel (a) forming a commanding peak. For the medium-weight vehicle category in panel (b), the percentage of two axle vehicles drops to about 50%, with three- to five-axle vehicles sharing another 30%, approximately. For the heavier vehicles, exceeding 260 kN, the five-axle configuration is the one most frequently observed, with a 74% relative frequency in panel (c) and 92% in panel (d). By comparison, the second most frequent appearance in panel (c) are four-axle vehicles, which amount to 18.7% of the observations, while for the exceptional vehicles in panel (d), they account for a little less than 5%.

**Fig. 6:**
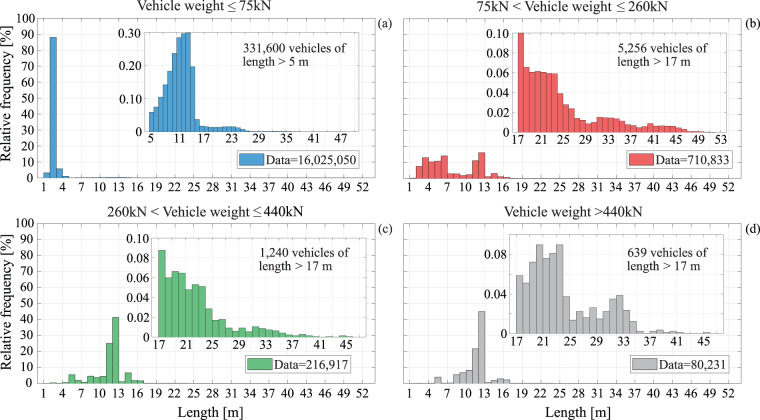
Vehicle length. (**a**) Relative frequency distribution of light-vehicle lengths; (**b**) relative frequency distribution of medium-vehicle lengths; (**c**) relative frequency distribution of heavy-vehicle lengths; (**d**) relative frequency distribution of lengths of vehicles with mass greater than 44 t.

**Fig. 7:**
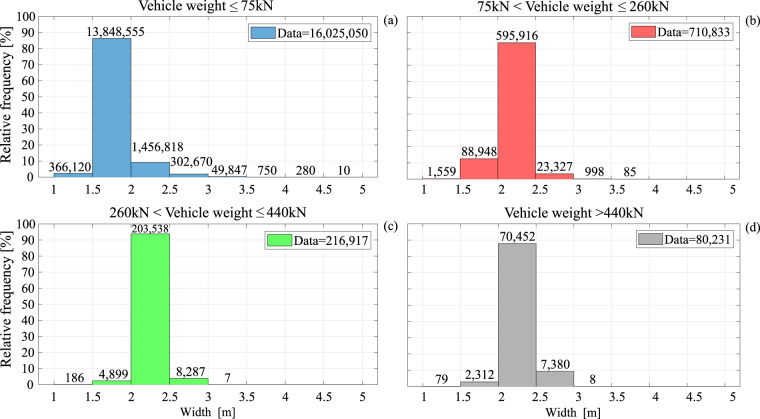
Distributions of vehicle width. Relative frequency distribution of (**a**) light-vehicle width; (**b**) medium-vehicle width; (**c**) heavy-vehicle width; (**d**) width for vehicles with mass in excess of 44 t.

Figure 6 shows the frequency distributions of vehicle length for the four weight categories. Recalling that the dataset contains no motorbike records, and that length is defined as the distance between the outermost axles, the minimum recorded length value is about 1.50 m. On the other hand, while a filtering restriction was imposed on the maximum number of axles, no further restriction was imposed for length, with some few vehicles exceeding 40 m. For example, the longest recorded vehicle falling within the records in panel (a), has a length of 47.3 m and weight of 68 kN, corresponding to a ten-axle vehicle with a 1.76 m width. Moving from lighter to heavier vehicle categories, that is from panel (a) to (d), mean length increases from 2.8 m to 7.8 m, 11.5 m, and 11.6 m, respectively. In Fig. 7, the frequency distributions of width conditioned by vehicle weight category are depicted. As expected, this data exhibits less heterogeneity than lengths and weights, with almost 90% of the light-vehicle observed data falling between 1.5 m and 2 m and more than 80% of medium and heavy vehicles between 2 m and 2.5 m. The largest recorded vehicle width was 4.7 m, falling under panel (a) jurisdiction, being a two-axle vehicle, 2.9 m long and weighing 20 kN.

**Fig. 8:**
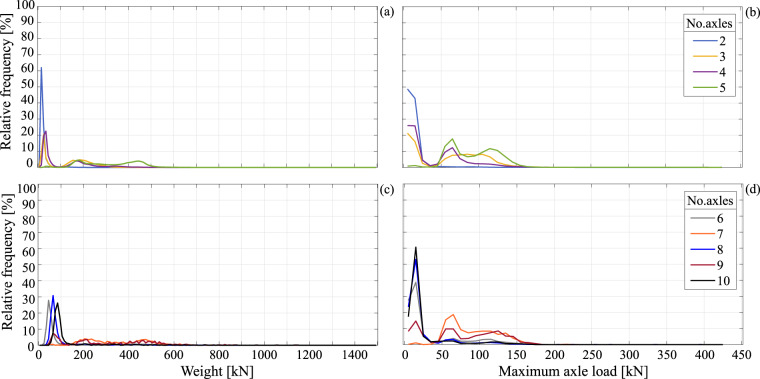
Relative frequency distributions given number of vehicle axles: (**a**) weight distributions, two to five axles; (**b**) highest axle load per vehicle, two to five axles; (**c**) weight, six to ten axles; (**d**) highest axle load, six to ten axles.

**Fig. 9:**
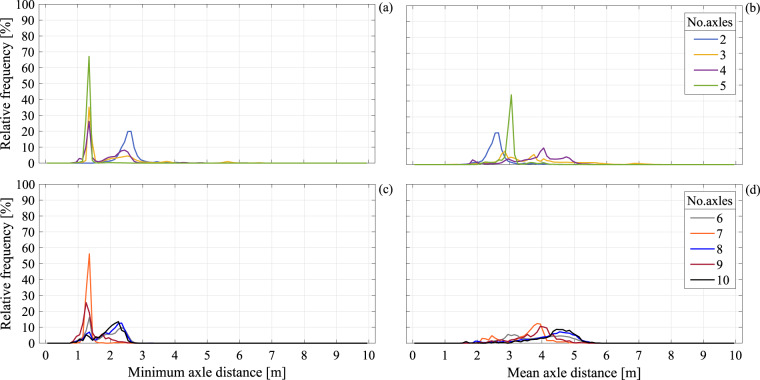
Relative frequency distributions given number of vehicle axles: (**a**) minimum axle distance distributions, two to five axles; (**b**) mean axle distance, two to five axles; (**c**) minimum axle distance, six to ten axles; (**d**) mean axle distance, six to ten axles.

As mentioned, another way to classify the vehicles is to stratify the data according to the number of axles. For example, Table 2 gives a breakdown of the number of observed vehicles per axle-number configuration, as well as the maximum recorded values of geometry and weight parameters, that is vehicle weight, highest axle load, length, and width. The frequency distributions of weight, highest axle load per single vehicle, minimum and mean distance between a vehicle’s consecutive axles, each for a given number of axles, are shown from Figs. 8, 9. Such conditional distributions can prove useful in cases where traffic simulation applications dictate a need to sample the joint distribution of vehicle geometry and axle-load parameters. To improve data visibility in these graphs, the upper two panels of each figure refer to vehicle axle numbers ranging from two to five, while information pertaining to vehicles with six to ten axles occupy the lower two panels.

**Table 2:** Number of records breakdown according to each vehicle’s number of axles and min/max recorded value intervals for each parameter and vehicle category.

No. axles	No. data and % of total		Maximum Weight [kN]	Maximum axle load [kN]	Length [m]		Width [m]	
					min	max	min	max
2	16,041,498	94.2%	740	400.0	1.50	10.00	1.0	4.7
3	144,015	0.8%	716	390.3	1.62	19.89	1.0	3.5
4	426,531	2.5%	940	429.8	2.82	25.12	1.0	3.3
5	384,836	2.2%	1278	398.4	4.06	28.66	1.0	3.2
6	30,601	0.2%	1480	383.4	6.63	32.37	1.0	3.1
7	1,117	0.007%	814	224.0	10.79	34.37	1.7	2.7
8	3,283	0.019%	1110	391.7	8.36	40.39	1.0	3.1
9	471	0.003%	872	176.5	14.72	42.93	1.1	2.5
10	679	0.004%	1148	161.6	13.97	51.53	1.4	2.8

In the same spirit of enhancing data visibility, in each panel, the frequency distribution histograms are represented by continuous lines, in lieu of the bars used in previous figures. The lines connect the relative frequency values at the abscissa of fixed-width bin centres, allowing the superposition of multiple distributions per panel. Figure 8 shows conditional frequency distributions for vehicle weight and highest axle load per vehicle, plotted at a step (bin width) of 10 kN. From the figure, it can be observed that, with the exception of two-axle vehicles, the distributions tend to be multimodal, an effect that is more pronounced for the highest axle load distributions.

Figure 9 shows the conditional frequency distributions of minimum and mean axle distance per vehicle category. Geometrical information such as the distance between consecutive axles can be important for determining the effect of traffic loads on bridges, as the concentration of forces on a region of the deck can aggravate structural demand in terms of stresses.

## Technical Validation

**Fig. 10:**
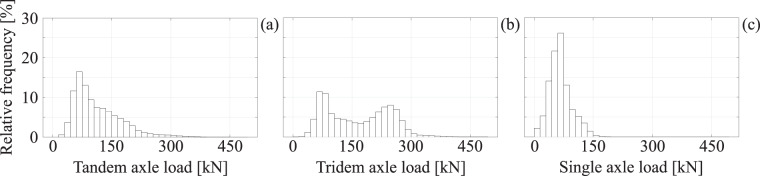
Marginal empirical distributions of heavier vehicle axle loads for (**a**) tandem axles, (**b**) tridem axles, (**c**) single axles.

As already mentioned in the Methods section, the recorded traffic data were subjected to a series of quality control verifications prior to inclusion in the provided dataset^12^. These included logical checks, such as verification that an axle load had been provided for each identified axle, that the sum of axle loads was summed up to the reported gross vehicle weight and that the sum of distances between consecutive axles were consistent with the overall length. Another indirect verification was to compare some of the empirical distributions with their counterparts published in the literature. Thus, the data were stratified in a different manner, that has found application in the construction of bridge load models in the past, by examining the load transferred by axle groupings of heavier vehicles, such as trucks. In this context, the recorded axle loads of all vehicles with a total weight in excess of 75 kN are divided into single axles, tandem axles and tridem axles, based on an inter-axle distance criterion whereupon axles less than 1.8 m distant from one-another are considered as part of a group and their loads are summed^23^. This operation is quite intuitive, as it can be expected that, given the total vehicle weight, the loads transferred through nearby axles are not independent. The marginal empirical distributions for the three axle groupings of all vehicles bar the light-vehicle category, are provided in the histograms of Fig. 10.

The figure shows that all three distributions exhibit one modal value at around 75 kN to 90 kN, but the tandem load distribution’s observed tail is far heavier than that of its single-axle counterpart, while the tridem axle load distribution is conspicuously bimodal. In fact, these two observations on the number and location of the (first) modal values, stemming from one year’s worth of traffic on the A3, are quite consistent with corresponding published distributions from WIM data recorded in France in the late eighties and mid-nineties^24,25^. This comparison is meaningful because the traffic data that were recorded on the French A6 highway in the late eighties and referenced above, were very influential on the development of the EN-1991-2 traffic load model for road bridges. On the other hand, a notable difference between the present dataset and these earlier data can be found in the maximum recorded values, with the maximum tridem axle load recorded among the recent Italian data being around 70 kN to 150 kN larger than those of the earlier datasets mentioned.

## Usage Notes

Over the past thirty-plus years, one of the salient applications of WIM data in the fields of structural and transportation engineering has been their use for the development and calibration of conventional code-mandated traffic loads to be used for the design and/or assessment of road bridges. Code design load values are based on underlying reliability criteria since they aspire to achieve predetermined levels of structural safety. In this context, bridge traffic loads should be calibrated to reflect rare actions, with a prescribed exceedance probability. At first glance, it may appear that vehicle weight and axle load statistics from WIM data, such as those presented, can be used for direct determination of such load values. However, this is not the case for two main reasons: first of all, typically available time intervals of continuous recordings are significantly inferior to the prescribed return periods of design actions for structural safety checks. Secondly, bridge actions, in terms of internal forces of structural elements such as flexural moment or shearing force, depend on the spatial disposition and concentration of axle loads, over a bridge’s deck, while WIM monitoring produces more point-like data.

In this light, WIM data are mainly exploited to set up and calibrate traffic simulations, aimed at alleviating the aforementioned limitations. Such simulations use WIM-based empirical distributions, or parametric models based on the same, by sampling circulating vehicle characteristics on multiple lanes, such as length, velocity, axle number, distances from one-another and axle loads, to create artificial records of traffic scenarios on bridges of interest. Because such simulations must, in principle, sample vehicle characteristics from the joint distribution of these parameters, the bar for the quality and completeness of the corresponding WIM data is set high. Case-in-point, while the EN-1991-2 load model calibration studies started off with availability of traffic monitoring and WIM data for several European highways, the conclusion was eventually reached that only a small fraction of those records contained sufficiently detailed vehicle geometry, speed, and axle load characteristics on multiple lanes, to be deemed an appropriate basis for meaningful traffic simulations^26^. Although the situation in Europe has since improved, this highlights the importance of traffic data availability carrying sufficient information to render them fit-for-purpose with respect to such engineering applications, as in the case at hand.

Other engineering applications that habitually exploit the availability of WIM data, are activities related to monitoring the behavior and durability or road pavement and in combination with structural health monitoring of bridges.

## Data Availability

The MATLAB dataset files, provided in addition to the coma-separated values format (available at figshare^12^) were prepared via MATHWORKS MATLAB® (release 2022a). The MATLAB code used to obtain the empirical distributions shown in this paper from the data, is freely available in the same repository as the data.
